# A new troglomorphic species of *Harmonicon* (Araneae, Mygalomorphae, Dipluridae) from Pará, Brazil, with notes on the genus

**DOI:** 10.3897/zookeys.389.6693

**Published:** 2014-03-14

**Authors:** Denis Rafael Pedroso, Renner Luiz Cerqueira Baptista

**Affiliations:** 1 Laboratório de Aracnologia, Departamento de Invertebrados, Museu Nacional / Universidade do Brasil (UFRJ). Quinta da Boa Vista, São Cristóvão, 20.940-040, Rio de Janeiro, RJ, Brazil; 2 Laboratório de Diversidade de Aracnídeos, Instituto de Biologia, Universidade do Brasil (UFRJ)

**Keywords:** Amazonia, biodiversity, cave, Diplurinae, Neotropics, taxonomy

## Abstract

A new species of *Harmonicon* F. O. Pickard-Cambridge, 1896 (Araneae, Dipluridae) is described, from a medium-sized lateritic cave in Parauapebas, Pará, Brazil. The male holotype and only specimen known of *H. cerberus* **sp. n.** was found near the entrance of Pequiá cave. This taxon is the fourth species described and the southernmost record for the genus. The new species displays some troglomorphic characteristics, such as reduction and merging of the posterior median and both pairs of lateral eyes and pale yellow to light brown coloration. Both characters are diagnostic when compared to the normal separated eyes and reddish to dark brown of other *Harmonicon* species. Other diagnostic characteristics are isolated, long, rigid setae distal to the lyra and the shape of the copulatory bulb. This is the second troglomorphic mygalomorph species from Brazil and the first from the Amazonian region.

## Introduction

*Harmonicon* F. O. Pickard-Cambridge, 1896 is a Neotropical genus of Mygalomorphae, belonging to the family Dipluridae. There are three described species from the Amazon region (Platnick 2014): *Harmonicon audeae* Maréchal & Marty, 1998 (males and females, from Sinnamary, French Guiana), *Harmonicon oiapoqueae* Drolshagen & Bäckstam, 2011 (males and females, from Saint Georges, French Guiana), and *Harmonicon rufescens* F. O. P.-Cambridge, 1896 (immature, from Santarém, Pará state, Brazil). This genus was removed from former synonymy with *Diplura* C. L. Koch, 1850 by Maréchal and Marty (1998), and it was recently placed in Diplurinae by Drolshagen and Bäckstam (2011), due to the presence of a lyra. In the family Dipluridae, this structure is known only in two other genera of that subfamily, *Diplura* and *Trechona* C. L. Koch, 1850.

The new species was found in the entrance of Pequiá cave, a medium-sized lateritic cave in the Floresta Nacional de Carajás, Parauapebas, Pará, Brazil. One of approximately 1,100 caves in iron ore deposits found in Carajás, Pequiá cave is situated at 06°05'15" S, 50°07'13"W (DMS), *circa* 427 m above sea level (IBAMA 2003, Piló and Auler 2009, IPHAN 2013). This cave has a projection of approximately 72 m, an L-shaped form, and contains a permanent water pool covered with guano (IBAMA 2003, Magalhães 2012, IPHAN 2013). Pequiá cave harbors important remains of earlier indigenous occupation in the Amazon region (IBAMA 2003, Magalhães 2012, IPHAN 2013).

There are several studies on the cave fauna of the iron ore cave region of Carajás (ex. Cunha et al. 2007, Pellegrini and Ferreira 2011, Prous et al. 2011). Several troglobitic species have been found in these iron ore caves, including a beetle and a centipede (Trajano and Bichuette 2010; Pellegrini and Ferreira 2011). There is a high potential of iron caves as habitat of troglobitic invertebrates in Brazil (Trajano and Bichuette 2010). As determined by Prous et al. (2011), there are an average of 2.5 troglobitic species in each cave with permanent water bodies in the Carajás region.

This is the second troglomorphic mygalomorph species from Brazil, but the first species from the Amazonian region. Recently, a troglobitic Theraphosidae was described from Bahia state (Bertani et al. 2013). Troglobitic or troglomorphic Dipluridae are common in subfamilies other than Diplurinae (ex. Euagriinae, see Coyle 1988), but the only other diplurine described solely from caves is *Linothele cavicola* Goloboff, 1994. This species lacks most of the modifications commonly associated with cave life, such as pigmentation and eye reduction, but displays elongated appendages, a reduced number of teeth on tarsal claw, and does not spin webs (Goloboff 1994).

## Methods

The color pattern was based on a specimen preserved in 75% ethanol. Observations, photographs and measurements were made with an Olympus stereoscopic microscope. Measurements are given in millimeters, unless otherwise noted. Cephalothorax length was measured from the posterior border to the anterior margin of the clypeus. Total length was measured from the posterior border of the anal tubercle to the anterior margin of the clypeus, not including the spinnerets. Each article of the pedipalp and legs was measured in retrolateral view, from the basal condylus to the distal one. Photographs were taken with a Sony Cybershot DSC-V1 camera attached to the stereomicroscope. The software package COMBINEZ, version COMBINEZP (Hadley 2013), was used to create composite images with extended depth of field. Geographical coordinates for localities were obtained from GEONAMES (2013). The distribution map was elaborated using ESRI ARCGIS 10 software.

The following abbreviations are used: ALE = anterior lateral eyes; AME = anterior median eyes; ITC = inferior (or unpaired) tarsal claws; PLE = posterior lateral eyes; PLS = posterior lateral spinnerets; PME = posterior median eyes; PMS = posterior median spinnerets; STC = superior (or paired) tarsal claws. Spines (or macroseta): ap = apical; p = prolateral; pld = prolaterodorsal; plv = prolateroventral; r = retrolateral; rld = retrolaterodorsal; rlv = retrolateroventral; v = ventral; MNRJ = Museu Nacional, Universidade do Brasil/Universidade Federal do Rio de Janeiro, Brazil.

## Taxonomy

### *Harmonicon* F. O. P. Cambridge, 1896

*Harmonicon* F. O. P.-Cambridge, 1896: 755; Maréchal and Marty 1998: 500; Drolshagen and Bäckstam 2011: 91; Platnick 2014.

#### Harmonicon cerberus sp. n.

http://zoobank.org/9AE08814-D3FA-4DCC-9F8E-1493D6CE7E13

http://species-id.net/wiki/Harmonicon_cerberus

Figs 1 –11 , Map 1 , Table 1

##### Diagnosis.

**Figures 1–5.**
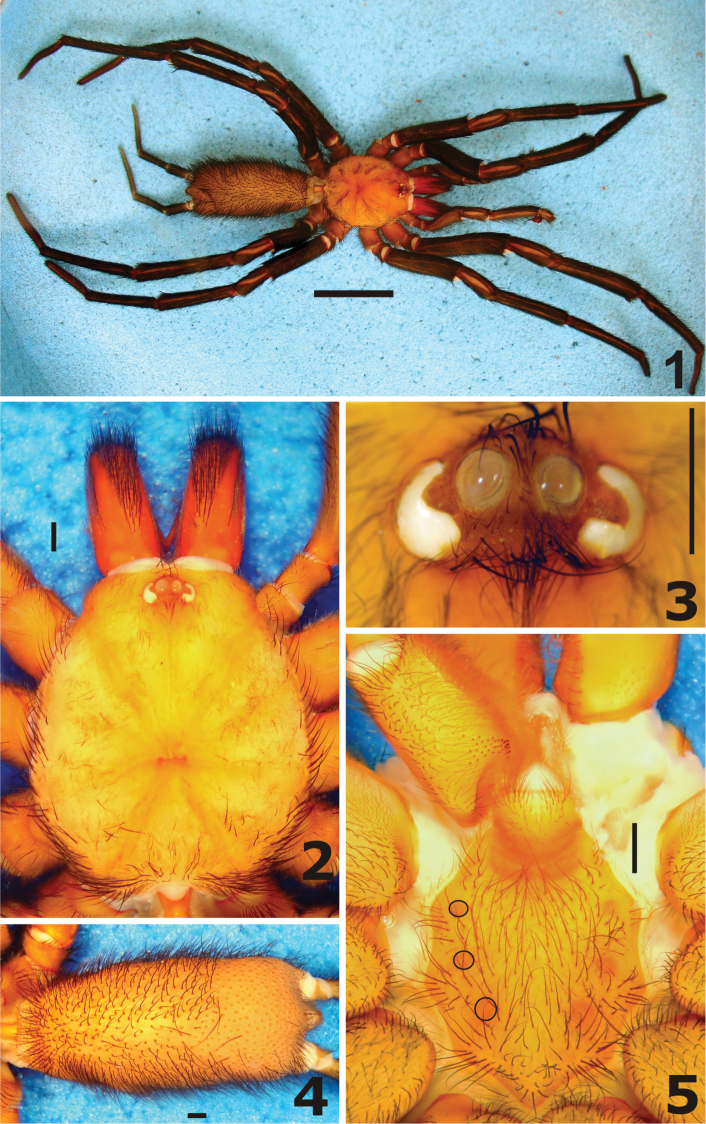
*Harmonicon cerberus* sp. n. Male holotype: **1** habitus **2** carapace, dorsal view **3** eyes, dorsal view **4** abdomen, dorsal view **5** sternum, ventral view.

**Figures 6–11.**
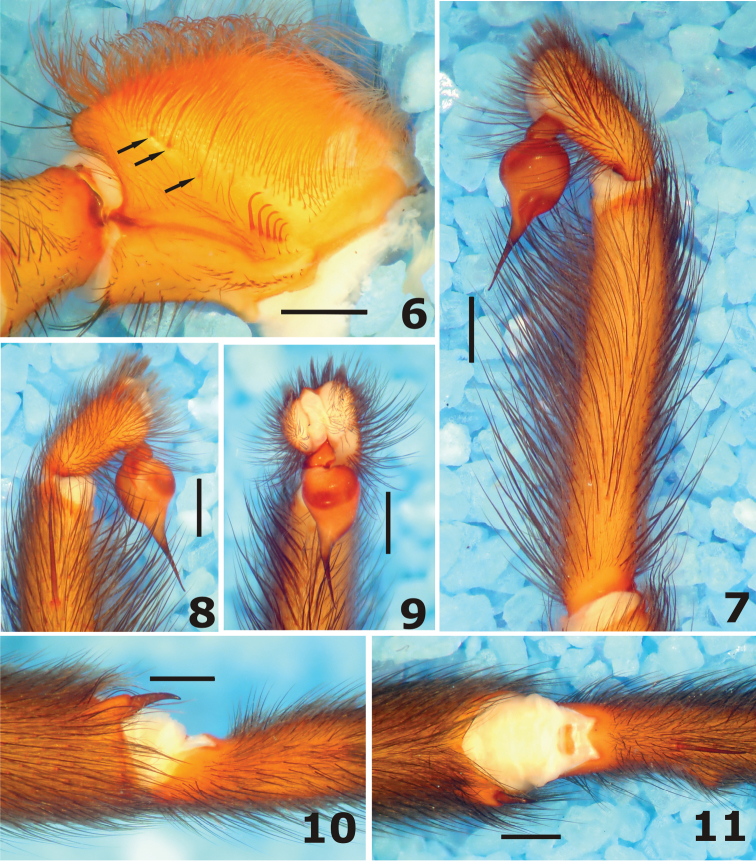
*Harmonicon cerberus* sp. n. Male holotype: **6** maxillae and lyra, ventral view **7** palp and bulb, retrolateral view; copulatory bulb **8** prolateral view **9** frontal view; tibia and metatarsus of leg I **10** retrolateral view **11** ventral view.

This species may be easily recognized by the fusion of the PME and all lateral eyes (Figs 1–3), elongated chelicerae, and pale coloration (Figs 1–2). Another diagnostic trait is the strongly thickened setae near the lyra (Fig. 6). In other *Harmonicon*, similar setae are found, but they are never so thickened. In prolateral view, the globose bulb, with a strong constriction around the basis of the embolus, resembles *Harmonicon oiapoqueae*, in contrast to the piriform bulb, regularly tapering toward the embolus, in *Harmonicon audeae*. The embolus is slightly longer than the bulb itself (ratio 1.3), similar to *Harmonicon audeae* (1.2), but shorter than in *Harmonicon oiapoqueae* (1.6).

##### Type material.

Male holotype from Brazil: *Pará*: Parauapebas, Floresta Nacional de Carajás, Pequiá Cave, near entrance, September 2003, Bittencourt, R. (MNRJ 04319).

##### Etymology.

The specific epithet “*cerberus*” is an apposition noun and a reference to the three-headed watchdog that guards the entrance to the underworld, the Hades, in Greek mythology.

##### Description.

Male (holotype, Figs 1–11). Measurements: carapace 10.7 long, 9.3 wide, chelicerae 4.4. Abdomen 14.5 long, 6.6 wide. Spinnerets: PMS 2.1 long, 2.0 apart; PLS, total length 18.1, basal article 4.9, middle 6.2, distal 7.0, 2.0. apart; respectively. Legs: see Table 1.

**Table 1.** *Harmonicon cerberus* sp. n. male holotype. Length of leg articles.

	Leg I	Leg II	Leg III	Leg IV
**Fe**	13.2	12.1	11.2	13.6
**Pa**	5.7	4.9	4.7	5.0
**Ti**	10.2	9.9	9.4	11.9
**Mt**	13.8	11.9	13.1	17.2
**Ta**	9.3	8.8	8.0	10.0
**Total**	52.2	47.6	46.4	57.7

**Carapace** (Figs 1, 2) length/width 1.15; flat, cephalic area slightly raised, thoracic furrows shallow and wide. Fovea: 1.0 wide; deep, straight. Carapace with many short, thin setae, interspersed with some longer and thicker setae; border with abundant long and thick setae pointing out, increasing in number towards posterior angles. Clypeus 0.3, frontal margin bearing 9 thick, long, erect setae (Fig. 3). **Eye tubercle** (Fig. 3) length 0.9, width 1.9, with sparse thin setae separating eyes of both sides and thicker, longer setae at the anterior and posterior borders. AME elliptical, with a milky lens, yellowish brown background, no retina or eye pigments visible. Left AME larger than right one. All other eyes (ALE, PME and PLE) fused in an asymmetrical, crescent shaped, lateral eye mass, with irregular borders, covered by a thin lens, with uniform white background. Right lateral eye mass thinner, shorter and with a more pronounced notch than the left one. Eye row curvature not definable, but AME anterior border a little advanced in relation to anterior border of lateral eye mass (Fig. 3). Right AME 0.3, AME–AME 0.3, right lateral eye mass 0.6 long. **Chelicerae** (Fig. 2) length/carapace length 0.41, 11 and 12 teeth on promargin, on the left and right chelicera, respectively. **Maxillae** (Figs 5, 6) length\width: 2.1. Cuspules: 36 spread over ventral inner heel. Lyra at the ventral side of the maxilla, asymmetrical, formed by 4–5 modified thick, long setae, increasing in size from basal to distal one, strongly curved at apical portion, apex just tapering to a point. Right lyra with just 4 setae, left lyra with 5 large setae and a very small, thinner basal one. Thick, erect, regularly curved setae (Fig. 6, arrow) placed distally and a bit internally in relation to lyra, in number of 4 at the right maxilla and 3 at the left one. Labium: length/width 0.8, no cuspules. Labio-sternal groove deep, with elongated sigilla. **Sternum** (Fig. 5) length (up to labium border) 5.3, width 4.7. Posterior angle in a blunt point, not separating coxae IV. Sigilla: three pairs, elliptical, increasing in size backwards, all far from margin by its own size. **Palp** long, without spines at retrolateral side, one prolateral spine at distal third of femur, 2 prolateral spines at tibia, distal one longer and thicker. Tibia: length 6.3, thin and long, with similar diameter throughout, length/width 9.1. **Leg** formula 4123. Legs covered with more abundant short, thin, horizontal black setae and with many longer, thicker, erect black setae. Leg I with modified tibia and metatarsus, forming a retrolateral clasping mechanism (Figs 10–11). Tibia I with a retrolateral distal spur (or apophysis) relatively long, somewhat curved, blunt, bearing a curved, pointed spine at tip. Metatarsus I with small retrolateral tubercle, situated distally to basis of first ventral spine. Tarsal trichobothria much longer than covering setae, placed in a row along the midline of dorsal face. Scopula undivided, covering distal half of metatarsus I and distal third of metatarsi II-III; all tarsi covered with scopula throughout length. All tarsi flexible, with abundant cracks. Spines: leg I: femur d2-2-0 left, d2-1-0 right, pld0-2-1 left, pld1-1-1 right, rld1-2-1 left, rld1-1-2 right; patella 0; tibia p1-0-1, r1-0-1 left, r1-0-0 right, v1-1-1ap (apophysis) left, v2-1-1ap (apophysis) right; metatarsus p0-1-0, v1-2-0 left, v1-2-1ap right; leg II: femur d2-2-0 left, d3-1-0 right, pld1-3-1 left, pld1-2-1 right; patella 0; tibia p1-1-1 left, p1-0-1 right, v2-1-1ap left, v1-1-1ap right; metatarsus pld0-1-0, v1-2-2ap; leg III: femur d2-1-0 left, d3-1-0 right, pld0-2-1, rld1-1-1 left, rld1-2-1 right; patella 0, tibia plv0-1-0 right, pld1-0-1 right, rld1-2-1, v2-2-2ap; metatarsus d2-1-1 left, d1-1-1 right, pld2-2-1 left, pld1-2-0 right, rld1-3-0 left, rld1-3-1 right, v2-1-3ap left, v1-1-2ap right; leg IV: femur d2-1-0, pld1-2-1 left, pld0-2-1 right, rld2-3-1 left, rld2-1-2 right; patella 0, tibia pld1-1-0 left, pld1-2-0 right, rld1-2-2(1ap) left, rld1-2-1 right, v2-2-2ap; metatarsus d1-1-1 left, d2-1-0 right, pld2-1-1 left, pld2-2-1 right, rld2-3-2 left, rld1-2-1 right, v2-1-2ap. Claws: ITC without teeth. Teeth at STC: leg I inner row 4–5, outer row 10–13; leg II inner row 4–5, outer row 10–12; leg III inner row 3, outer row 9–11; leg IV inner row 2–3, outer row 7–10. **Bulb** (Figs 7–9) globose, with moderately long embolus, a little longer than basis (ratio 1,3). Bulb with prolateral face convex, gently and uniformly curved, retrolateral face convex at basis and concave at end portion, due to an abrupt curve, forming a strong constriction of bulb near basis of embolus. Embolus with a broad basis, regularly tapering to tip, both in prolateral and retrolateral views. In frontal view, embolus placed at prolateral margin of bulb, slightly curved initially, straight through most of its length, with apex bent retrolaterally (Fig. 9). Also, there is a strong bulge of bulb near embolus basis at same view.

**Color pattern (in 75% ethanol**). Carapace pale yellow, with thoracic furrows and cephalic area just a little darker, with orange hue, chelicerae light reddish brown, labium, sternum and leg coxae grayish yellow, sigillae darker, with orange hue, other leg articles brownish yellow. Abdomen grayish yellow, with abundant long, thick black hairs.

##### Distribution

**Map 1.**
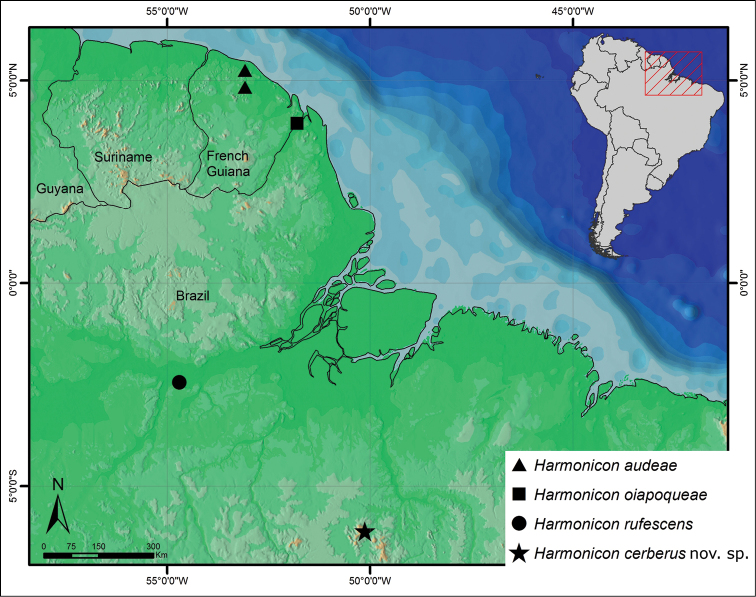
Distribution map with published records for *Harmonicon* species.

(Map 1). know only from type locality, in southern Pará state, Brazil.

### Remarks

**Troglomorphism.** The pale body, fused and reduced eyes and the elongated chelicerae (Figs 1–3) observed in *Harmonicon cerberus* sp. n. seem to be troglomorphic. *Harmonicon* species display usually a highly contrasting color pattern, with a reddish carapace, a black, dark brown or reddish brown abdomen and dark brown legs. Eyes of *Harmonicon* tipically are without modification, with two large AME, surrounded by distinct ALE, PME and PLE. The retina and pigmentation of the AME are easily seen through the clear crystalline lens. In contrast, the AME of the new species bears a milky crystalline and no trace of a retina or pigmentation are visible behind it. The ALE and AME of the other *Harmonicon* bear distinct crystalline and spherical lenses. However, ALE, PME and PLE of *Harmonicon cerberus* sp. n. are fused, forming an irregular and asymmetrical white macula, covered by a shallow and irregular lens (Fig. 3). To the best of our knowledge, the crescent shaped lateral eye mass is a rare character in spiders. The elongated chelicera (more than 40 % of the length of carapace) is longer than in other species of the genus. The legs and spinnerets are also elongated, but males of *Harmonicon audeae* and *Harmonicon oiapoqueae* also have similar long legs. An additional possible troglomorphic character is the elongated trichobothria at the leg tarsus. In comparison to other *Harmonicon* species, the trichobothria of the new species are approximately twice as longer than the covering hairs and clearly visible in lateral view. Furthermore, *Harmonicon cerberus* sp. n. seems to bear more unequivocal troglomorphic characteristics compared to *Linothele cavicola*, the only other cave inhabiting Diplurinae (Goloboff 1994).

This spider may be a troglobitic species, despite the small dimensions of Pequiá cave. Several other troglobitic species have been collected in some of the more than a thousand iron ore caves in Carajás (Piló and Auler 2009, Trajano and Bichuette 2010, Pellegrini and Ferreira 2011). Most of those caves are reduced in size (horizontal projection 20-30 m), but they may be connected by small conduits, due to the porosity and spongiform nature of the iron ore deposits of the area (Piló and Auler 2009).

Pequiá cave and its surroundings have been thoroughly investigated recently (Pedroso, pers. obs.). However, no additional specimens of *Harmonicon* have been found. On the other hand, only the entrance and the beginning of the lateral tube were investigated (around 1/3 of the cave), as there is a large water pool mixed with abundant guano. Unfortunately, the exploration of the terminal portion of the lateral tube of Pequiá cave needs special equipment, such as floaters. This portion appears to be the best candidate for new attempts to find additional specimens of *Harmonicon cerberus* sp. n., as it is farthest away from the mouth and the darkest area of the cave. Other possible areas for exploration are the numerous caves near Pequiá cave, specially Gavião cave, a larger cave situated *circa* 4 km from Pequiá cave (Magalhães 2012).

**Notes on *Harmonicon*.** In their paper on the revalidation of *Harmonicon*, Maréchal and Marty (1998) proposed a series of diagnostic characters for the genus: leg I longer than IV (leg formula 1423), contrasting with formula 4123 found in *Diplura* and other Diplurinae; legs longer and thinner than in *Diplura*; metatarsus I of males without the prolateral knob found in other Diplurinae; lyra formed by only 5 setae, with a flattened and curved tip, compared to *Diplura*, where it presents more setae, with a different tip.

The diagnosis of *Harmonicon* is not so clear-cut. Regarding the leg formula, the female of *Harmonicon audeae* itself, the species described by Maréchal and Marty (1998), has leg formula 4123, as does the female of *Harmonicon oiapoqueae* (Drolshagen and Bäckstam 2011). The males of *Harmonicon audeae* and *Harmonicon oiapoqueae* do have a leg formula of 1423. On the other hand, the legs of the male holotype of *Harmonicon cerberus* sp. n. follow the formula 4123. Furthermore, most Diplurinae males have longer anterior legs, following the formula 1423 (and even 1243), so this character is not reliable for diagnosing *Harmonicon*.

Another inconsistent character is the absence of the prolateral knob of male metatarsus I. Both *Harmonicon oiapoqueae* and *Harmonicon cerberus* sp. n. present the cited knob, so its absence may be an autapomorphy of *Harmonicon audeae*.

Considering the lyra, the number and shape of the setae are not consistent throughout Diplurinae. Some *Diplura* species we have examined have lyra with just a few setae (down to 2), sometimes with tip curved and somewhat flattened. The setae tip though is not as curved and flattened as in *Harmonicon*. Again the number of setae is not a reliable character for *Harmonicon*, as already suggested by Drolshagen and Bäckstam (2011).

Additional diagnostic characters for *Harmonicon* were proposed by Drolshagen and Bäckstam (2011): presence of dense scopula in more than the apical third of pedipalpal tarsus, presence of scopula in the apical third of most leg metatarsi, and leg tarsi “pseudosegmented” (instead of showing only a few cracks). The dense scopula in legs is similar to that found in *Trechona*, where it is very dense. In *Diplura*, the scopula is thin, sometimes not conspicuous at all.

The presence of additional, rigid, and somewhat thickened, setae situated distally to the lyra (Fig. 6) may be diagnostic for *Harmonicon* or at least most of its species. Besides *Harmonicon cerberus* sp. n., these setae are found in specimens of *Harmonicon rufescens* from Altamira (unpublished data), *Harmonicon oiapoqueae* (see a picture on the website of Drolshagen 2013) and several undescribed species from Brazil. However, an undescribed *Harmonicon* from Mato Grosso lacks these setae altogether. No such setae were found in all other species of Diplurinae genera we examined.

Some additional characters may prove to be diagnostic, at least in relation to *Diplura*. The longer tibia of the male pedipalp may distinguish *Harmonicon* from *Diplura* species, in which tibia are usually short and stout. Long and relatively thin tibia are found also in the diplurines *Trechona* (ex. Pedroso and Baptista 2004, Pedroso et al. 2008) and *Linothele* Karsch, 1879 (ex. Paz and Raven 1990). The copulatory bulb of males of most *Harmonicon* is also remarkably similar to *Trechona* species (ex. Pedroso and Baptista 2004, Pedroso et al. 2008), with an enlarged basal portion and an elongated embolus.

The legs and palp with denser and longer scopula, the longer tibia of the male palp and the flexible, highly cracked tarsi may indicate a closer relationship between *Harmonicon*, *Trechona*, and perhaps *Linothele*. Another common trait of these genera is the increased body size, compared to *Diplura*. Compared to *Trechona*, some easily seen diagnostic characters of *Harmonicon* are the lyra composed by less than ten setae (vs. complex lyra, with more than 50 setae in several layers) and the absence of the chevron pattern on the abdomen (vs. presence).

All the published records for described *Harmonicon* species are located in the northeastern Amazon region, from northern French Guiana to central Pará state, Brazil (Map 1). The type locality of *Harmonicon cerberus* sp. n. is the southernmost record for the genus, located in southern Pará state. However, we examined specimens of several *Harmonicon* species covering a much larger area, ranging from Peru in the west, to states in northeastern (Bahia, Ceará) and central (Mato Grosso, Goiás) regions in Brazil.

## Supplementary Material

XML Treatment for Harmonicon cerberus
